# Designing a blockchain technology platform for enhancing the pre-exposure prophylaxis care continuum

**DOI:** 10.1093/jamiaopen/ooae140

**Published:** 2024-12-19

**Authors:** Anjum Khurshid, Daniel Toshio Harrell, Dennis Li, Camden Hallmark, Ladd Hanson, Nishi Viswanathan, Michelle Carr, Armand Brown, Marlene McNeese, Kayo Fujimoto

**Affiliations:** Harvard Medical School and Harvard Pilgrim Health Care Institute, Boston, MA 02215, United States; Harvard Medical School and Harvard Pilgrim Health Care Institute, Boston, MA 02215, United States; University of Texas at Austin-Dell Medical School, Austin, TX 78712, United States; Center for Dissemination and Implementation Science, Northwestern University Feinberg School of Medicine, Chicago, IL 60611, United States; Division of Disease Prevention and Control, Houston Health Department, Houston, TX 77054, United States; University of Texas at Austin-Information Technology Services, Austin, TX 78701, United States; University of Texas at Austin-Dell Medical School, Austin, TX 78712, United States; Division of Disease Prevention and Control, Houston Health Department, Houston, TX 77054, United States; Division of Disease Prevention and Control, Houston Health Department, Houston, TX 77054, United States; Division of Disease Prevention and Control, Houston Health Department, Houston, TX 77054, United States; Department of Health Promotion & Behavioral Sciences, School of Public Health, The University of Texas Health Science Center at Houston, Houston, TX 77030, United States

**Keywords:** HIV prevention intervention, PrEP care continuum, blockchain technology, self-sovereign identity, verifiable credentials

## Abstract

**Objectives:**

Pre-exposure prophylaxis (PrEP) is a key biomedical intervention for ending the HIV epidemic in the United States, but its uptake is impeded by systemic barriers, including fragmented workflows and ineffective data coordination. This study aims to design PrEPLinker, a blockchain-based, client-centered platform to enhance care to address these challenges by improving care coordination and enabling clients to securely manage their identity and PrEP-related data.

**Materials and Methods:**

Using Houston, Texas, as a use case, we conducted a needs assessment with PrEP collaborators to evaluate existing workflows and identify barriers in the PrEP care continuum. Based on these findings, we designed PrEPinker, a blockchain-based identity framework and digital wallet using self-sovereign identity and verifiable credentials (VCs). These features enable clients to securirely control their identity data and facilitate efficient, privacy-serving data sharing across PrEP service points, such as community testing sites, clinics, and pharmacies.

**Results:**

The needs assessment identified significant gaps in data exchange for PrEP referrals and follow-up appointments. In response, PrEPLinker was designed to incorporate decentralized identifiers—unique, secure digital identifiers that are not linked to any centralized authority—and VCs for ensuring seamless transfer of digital medical records. Preliminary usability testing with 15 participants showed that over 70% rated the interactive design positively, finding it easy to use, learn, and navigate without technical support. Additionally, more than 80% expressed confidence in using the blockchain based platform to manage sensitive health information securely.

**Discussion and Conclusion:**

Blockchain technology offers a promising, client-centered solution for addressing systemic barriers in PrEP care by improving data cordination, security, and client control over personal health information. The design of PrEPLinker demorates the potential to streamline PrEP referrals, follow-up processes, and data managent. These advancements in data coordination and secruity could improve PrEP uptake and adherence, supporting efforts to reduce HIV transmission in Houston and beyond.

## Introduction

According to the Centers for Disease Control and Prevention (CDC), 1.2 million people aged ≥13 are living with HIV at the end of 2022, and 12.8% are living with undiagnosed HIV infection.^1^ Furthermore, there is a disproportionate burden of this infection on certain subpopulations, particularly racial and ethnic minorities and sexual minority men (SMMs).^1–4^ In response, the *Ending the HIV Epidemic in the US (EHE)* initiative started with the mission of decreasing new HIV infections by 90% by 2030. While advances in antiretroviral treatment have improved the life expectancy of people with HIV, a key strategy of EHE is to prevent the acquisition of HIV with increased use of pre-exposure prophylaxis (PrEP) among those at risk for HIV.^5^^,^^6^ However, despite the demonstrated effectiveness of PrEP in preventing HIV,^7^^,^^8^ the uptake of PrEP remains low, especially among disproportionately affected populations such as SMM, people of color, and those residing in under-resourced areas.^9^^,^^10^

A significant barrier to PrEP uptake is the lack of a streamlined and accessible care delivery system for navigating and monitoring PrEP services, particularly in high-need communities where social and structural determinants of health further complicate access.

Blockchain technology offers a promising solution to address these challenges by providing a secure, decentralized system for managing health data. Self-sovereign identity (SSI) is a key feature of this approach.^11–13^ It allows individuals to control and manage their own identity data without relying on centralized authorities. This is achieved through decentralized identifiers (DIDs)—unique digital identifiers that are not linked to any centralized entity—and verifiable credentials (VCs), which are secure, cryptographically verifiable digital certificates that enable the sharing of necessary information while maintaining privacy and confidentiality. These technologies provide users with control over their data, facilitating consent management and improving trust in data sharing processes.

As of 2020, Houston, Texas, has a greater burden of new HIV infections than both the state of Texas and the United States, with 67% of people with new infections being African American or Latinx people.^14–16^ In Texas, low rates of PrEP-eligible candidates are being prescribed PrEP, following a nationwide PrEP disengagement trend due to barriers such as distrust of the healthcare system, HIV stigma, and lack of client awareness or access.^17^^,^^18^

The fragmented healthcare model in the United States, coupled with the social stigma associated with the use of PrEP and HIV services, poses challenges in developing effective population- and community-level strategies to improve PrEP services. Technological innovations such as blockchain provide an opportunity to disrupt some of the broader social and structural determinants of health that traditional policy and operational interventions cannot easily address. Blockchain technology^19^ is particularly promising for improving PrEP referral coordination by building a collaborative peer-to-peer (P2P) network as part of a community-wide IT infrastructure. This network eliminates bottlenecks in information transfer, streamlines referrals, and enhances the coordination of the PrEP care delivery ecosystem.^20^

One of the unique advantages of blockchain lies in its ability to focus on the individual as the source of data exchange, bypassing the intermediaries of service providers. Furthermore, blockchain technology is well suited for trusted and immutable end-to-end tracking of healthcare services and products, as demonstrated in pharmaceutical supply chain management.^21^ It is built on a secure, encrypted P2P network that removes the need for third-party data intermediaries. In this model, clients control their data, which are securely transferred between them and their intended recipients, such as doctors or pharmacies, ensuring privacy and confidentiality while building trust.

Our prior research has explored the potential of blockchain technology and SSI for establishing a trusted data-sharing environment for vulnerable populations, including the homeless.^22^^,^^23^ In this context, blockchain uses a digital distributed ledger, creating a trusted network of participating health organizations. The distributed and immutable nature of blockchain technology provides a secure and transparent client-controlled system for recording and sharing data,^24^ which is highly beneficial in healthcare.^25^ SSI, which uses DIDs and VCs, allows individuals to share only the minimal, cryptographically verifiable information necessary. This approach provides clients with control over their data, enables consent management, and ensures privacy and confidentiality.^13^

## Objective

Recognizing the potential benefit of blockchain technology in addressing some of the information and data-related gaps in healthcare, our research team, in partnership with the Houston Health Department (HHD), conducted a quality improvement study to assess the applicability of blockchain technology for secure client navigation and data sharing and monitoring between referring service agencies and PrEP providers.

Our objective was to identify and systematize the existing PrEP data workflow within the local care delivery ecosystem—examining key points of service, such as community HIV testing, PrEP clinics, and pharmacies—to inform the design of a blockchain-based system referred to as PrEPlinker, which could bridge gaps in care coordination. The integration of this technology could optimize PrEP referrals, data monitoring, and care delivery, thus contributing to the eventual goal of EHE by significantly reducing the incidence of new HIV infections. This could further help address health inequities on the PrEP care continuum. This article presents our proposed design for the PrEPLinker system, a secure, client-centered care coordination platform that leverages blockchain technology, and discusses the potential benefits and challenges of our design. Our discussion will be framed within the context of improving PrEP navigation and data monitoring in Houston, Texas.

## Materials and methods

### Project workflow

Between Fall 2021 and Spring 2024, our research team conducted the study in 4 phases: planning, discovery, design, and (preliminary) evaluation.

#### Planning phase—Literature review 

During the planning phase, we reviewed the literature and engaged in in-depth discussions with a range of experts from various fields, including HIV surveillance, data security, and HIV prevention and care, as well as front-line staff from the HHD and the local EHE initiative. Our objective was to gain a better understanding of the barriers that hinder effective data coordination within the local PrEP care delivery ecosystem and to clarify the deliverables of the study.

#### Discovery phase—PrEP workflow analysis

During the discovery phase, we identified key informants and community collaborators through convenience sampling^26^ of existing community–research partnerships. Eligible participants were recruited via email, referrals from other participants, and announcements at meetings hosted by local HIV prevention working groups in Houston. We subsequently interviewed these individuals to understand diverse perspectives of processes within the PrEP care delivery system in Houston. This phase also involved site visits to local providers, nonprofit community-based organizations (CBOs) providing navigation and intake services, and EHE planning partner organizations or bodies. Additionally, we engaged with data scientists and epidemiologists at the HHD to understand their perspective of the existing process. The research team analyzed sample forms related to client intake (demographics, financial, and insurance information), PrEP referrals, risk assessment and serology results, PrEP medications, and metrics reported to the HHD. The discovery phase helped identify specific data elements and documents needed for beneficiary credentials by PrEP service providers.

We conducted 12 key informant interviews with local PrEP collaborators to assess their current workflows and barriers experienced in delivering or receiving PrEP services. During the interviews, our research team introduced the conceptual design and workflows of the PrEPLinker system to our participants. Our discussions focused on topics with prioritized questions for each collaborator related to current workflow issues within the PrEP care delivery system, PrEP-provider data needs and forms, data reported to the local health department or other funders, challenges with the coordination of PrEP services, and perceived key benefits and barriers to the implementation of the PrEPLinker workflow and design. Our interviewees included PrEP providers, HHD staff, and contractors involved in PrEP navigation and front-line PrEP navigators, data scientists, and health information exchange experts in Houston. An interview moderator followed a guide (available in Table S1), and responses were collected via interview notes. Generalized lessons and themes were identified using thematic analysis of participant responses.

#### Design phase—PrEP solution architecture

The design phase used insights and details from prior phases to design the PrEPLinker platform, adapting a system called MediLinker that was previously designed for the purpose of managing client identity and consent for persons experiencing homelessness in Austin, Texas.^23^^,^^27^^,^^28^ TestLinker, which is a separate proof-of-concept blockchain system, is designed to manage HIV testing status (P30AI161943). A summary table that compares these different platforms (MediLinker, TestLinker, PrEPLinker) is available in Table S2.

#### Evaluation phase—Usability testing

We conducted preliminary usability testing of the PrEPLinker framework with 15 public health students and employees representing a general user audience. Focusing on the first step of the PrEP care continuum, HIV testing, which represents the initial client interaction with the system, we presented participants with a visual mock-up of PrEPLinker; this mock-up is called TestLinker and is specific to HIV testing. As the first step in PrEPLinker, TestLinker operates within an HIV-status-neutral framework to confidentially exchange HIV test results with authorized entities in the PrEP referral system. Following the initial testing phase, the broader PrEPLinker system manages the remaining stages of the prevention workflow for clients on PrEP.

After the participants were oriented to the PrEPLinker framework (wireframe designs are available at https://gitpapl1.uth.tmc.edu/fujimoto-lab-uthealth-hhd/blockchain), we administered a quantitative survey to assess their user acceptability of using blockchain technology to manage sensitive HIV-related healthcare data throughout the PrEP care delivery process (survey items are provided in Table S3). This preliminary usability test provided insights into user perceptions and potential challenges in adopting blockchain-based systems for HIV prevention and care. Additional details about the evaluation phase data and methods can be found in the Supplemental Materials. The institutional IRB approved the data collection for this study (HSC-SPH-22-0567).

## Results

### Planning and discovery

**Figure 1.**
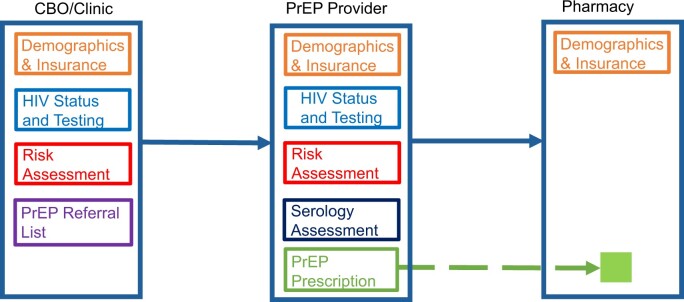
PrEP care continuum and client journey. According to local PrEP providers, the PrEP clients’ clinical journey can start with initial HIV testing at a community-based organization (CBO) or clinic where demographics are recorded and where HIV testing and risk assessments are performed. Once PrEP eligibility is determined, the client is referred to a PrEP provider for further assessment, and prescriptions are prescribed. A PrEP pharmacy dispenses the PrEP medication. Challenges such as duplicate information requests and redundant testing during care transitions can occur.

The planning and discovery phases resulted in the identification of specific data elements that are needed for the PrEP delivery system to function in a coordinated fashion. From the perspective of key informants, a potential PrEP client’s clinical journey typically starts at a CBO or clinic for HIV testing, where intake, HIV status determination, and risk assessments are performed. Once PrEP eligibility is determined, the client is referred to a PrEP provider for further assessments, and prescriptions are issued and then dispensed at a pharmacy. Through this care continuum, the PrEP client may have to provide the same information or perform duplicate tests during transitions of care (Figure 1).

These data elements were derived by comparing intake and assessment forms, referral formats used by clinical staff, and test results reported as required eligibility criteria. On the basis of these details, the research team developed the type of verifiable information or credentials that are exchanged among different users of the PrEP delivery system and the specific elements that describe each of those VCs. We also identified a need to close the loop of data exchange among various users of the PrEP delivery system to ensure effective coordination among providers by seamlessly sharing relevant information. PrEPLinker enables secure, real-time sharing of verified PrEP records through advanced encryption. This reduces fragmentation in care and potentially increases client retention during transitions between services by allowing providers to access the most updated client information and coordinate follow-up.

From our analysis, we designed 5 VC types: (1) health ID and insurance, (2) HIV serostatus, (3) PrEP referral data, (4) risk assessments, and (5) PrEP medication credentials. The data elements of each credential type follow the national standards of interoperability for clinical data (known as the HL7 FHIR) to provide reliable exchange of data between PrEPLinker and the provider’s electronic medical or health records. Most of the personal information in these VCs is provided by the client as self-reported attributes, whereas important clinical details are filled in by clinic staff in the current system and verified with trusted documents such as government-issued IDs, insurance cards, clinical forms, or lab results. Details of the results are shown in Table 1.

**Table 1.** PrEPLinker credentials and their attributes.

Credential type	Attributes	Verification process	Data entry
Health ID and Insurance	Preferred name	Self-reported	Client
	Client legal name	Government-issued ID	Client
	Given	Government-issued ID	Client
	Family	Government-issued ID	Client
	Other previous legal name	Self-reported	Client
	Date of birth	Government-issued ID	Client
	Phone number	Self-reported	Client
	Current street address	Government-issued ID	Client
	Last known address (type)	Self-reported	Client
	Zip code	Government-issued ID	Client
	City	Government-issued ID	Client
	State	Government-issued ID	Client
	Preferred language	Self-reported	Client
	Sex assigned at birth	Self-reported	Client
	Gender identity	Self-reported	Client
	Race	Self-reported	Client
	Ethnicity (Hispanic/Non-Hispanic)	Self-reported	Client
	Primary care doctor (Y/N)	Self-reported	Client
	Employment status (Y/N)	Self-reported	Client
	Insurance status (Y/N)	Insurance	Client
	If yes (Private, Harris Health Gold Card, Medicare, Medicaid, Other)	Insurance	Client
HIV Serostatus	HIV serostatus	Lab test report	Clinic staff
	Testing date	Lab test report	Clinic staff
	Test name	Lab test report	Clinic staff
	Test location	Lab test report	Clinic staff
PrEP Referral	Date of referral	Referral form	Clinic staff
	Referring agency	Referral form	Clinic staff
	Referring physicians	Referral form	Clinic staff
	PrEP user referred to	Referral form	Clinic staff
Risk Assessment	For the past 6 months, do you have sex with men, women, or both? (Men, Women, Men and Women, Trans-Male to Female, Trans-Female to Male, Unknown, Declined)	Self-reported	Client
	During past 12 months, how often do you use condoms during sex? (Always, Sometimes, Never)	Self-reported	Client
	In past year, have you been tested for Syphilis? (Y/N/Decline) If yes, Positive, Negative, Unknown	Self-reported	Client
	In past year, have you been tested for Gonorrhea? (Y/N/Decline) If yes, Positive, Negative, Unknown	Self-reported	Client
	In past year, have you been tested for Chlamydia? (Y/N/Decline) If yes, Positive, Negative, Unknown	Self-reported	Client
	In past year, have you been tested for Herpes? (Y/N/Decline) If yes, Positive, Negative, Unknown	Self-reported	Client
	In the past 12 months, have your partner(s) been diagnosed with any of the following: HIV, Syphilis, Gonorrhea, Chlamydia, Trichomonas, genital warts, or genital herpes? Yes, No, I do not know, Declined	Self-reported	Client
	In the past 12 months, have you had unprotected sex to get drugs or money? Yes, No, I do not know, Declined	Self-reported	Client
PrEP Medication	PrEP medication (ex: Truvada, Descovy, Apretude)	PrEP prescription	Clinic staff
	Prescription issued date	PrEP prescription	Clinic staff
	Prescription fulfilled date	PrEP prescription	Clinic staff

### Design

On the basis of in-depth interviews with key informants and site visits, we mapped out the data workflow for PrEP services in the Houston area. Combining our findings with some of the unique advantages of blockchain technology for sharing validated data, we developed the design of the PrEPLinker system. Different roles are assigned in the system on the basis of the role of entities in issuing, validating, or using identity credentials. ISSUERS creates digital VCs on the basis of physical records such as government-issued IDs or insurance cards. The issued VCs are held on the HOLDER’s digital wallet and sharable with future PrEP clinics or providers, who act as VERIFIERS.

Clients and organizations interact with their wallet via a mobile or web application. We envisioned that CBOs or health services organizations would serve as ISSUERS of the data related to health ID, insurance status, HIV status, risk assessments, and PrEP referral. The CBOs are typically the client’s first contact for HIV testing within the PrEP linkage and care continuum. The data related to these individual features captured by the CBOs during intake are stored as VCs to be held by the client in their dedicated, secure blockchain wallets. The client is the HOLDER of the information or VCs.

The concept of the wallet is not different from the digital wallets used by Apple Pay, Google Pay, and other services through mobile phones. The client can use their mobile devices to share their digitally trustable VCs, including health ID, HIV status, risk assessment, and PrEP referrals to their PrEP provider, who may use this information to evaluate the eligibility of the client for the PrEP program without having to request that information from other providers or organizations, which is usually an administrative time-consuming step.

A unique feature of PrEPLinker is its shift from centralized trust, as commonly found in large multinational big tech corporations, to a decentralized trust model. This model relies on a consensus mechanism governed by a P2P network. There are 2 key aspects of trust in the blockchain-based PrEPLinker system: first, the trust by clients that their information will not be used without transparency and their consent, and second, the trust by providers that they have access to updated information from all sites involved in the client’s care. The PrEPLinker system is designed to provide both levels of trust through immutable, secure, and decentralized blockchain technology.

**Figure 2.**
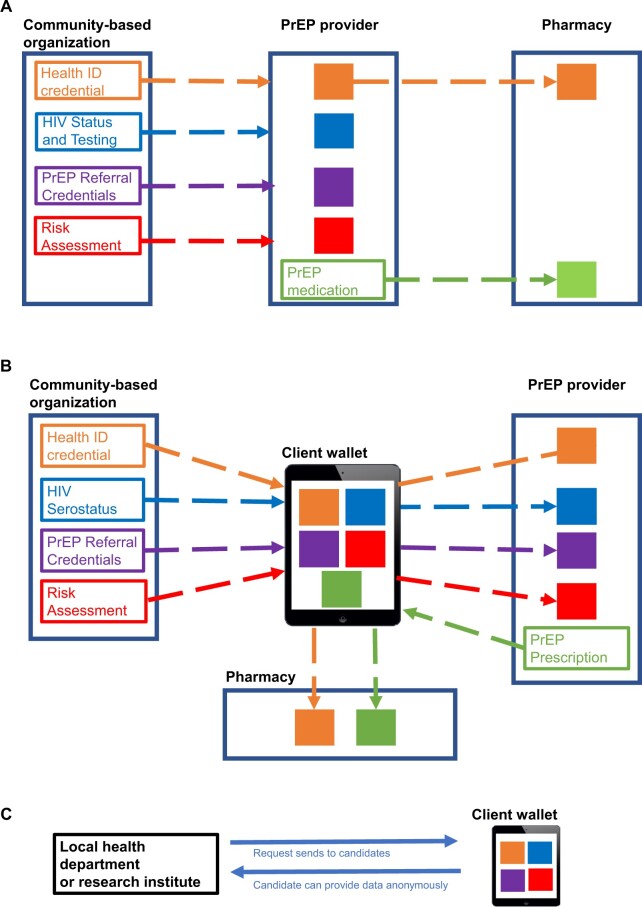
PrEPLinker data workflow. (A) PrEP clients typically begin prevention services at community-based organizations (CBOs) or health services organizations followed by referrals to a PrEP provider and prescription for fulfillment at a pharmacy. CBOs can issue health IDs, HIV status and testing, PrEP referrals, and risk assessment verifiable credentials (VCs) and then present 2 future PrEP providers. The PrEP provider can issue a PrEP medication VC for future presentation at a pharmacy. VC issuance is represented by rectangles, and VC issuance is represented by a solid square. (B) Each credential is held in the client’s PrEPLinker wallet and presented to future PrEP services via a mobile application. (C) Local health departments or research institutes can send requests to each client for their consent to share their PrEPLinker data for studies or services. If consent is given, the requested dataset can be sent anonymously and securely via the PrEPLinker mobile application.

Using blockchain technology’s immutable data characteristics, the minimum amount of information required to prove the client’s adulthood or HIV serostatus can be shared without revealing unnecessary personal information, a concept described as zero-knowledge proof in blockchain technology.^29^ On the basis of the PrEP course, a provider can issue a PrEP medication order in the form of a VC for fulfillment at a pharmacy. This information is shared with the client as well as the pharmacy via the PrEPLinker platform on the P2P network. At the pharmacy, the client can share their Health ID VC to verify identity and their PrEP medication prescription for fulfillment. Once fulfilled, the pharmacist can reissue the credential detailing the filled medication to share back with the client as well as the provider (Figure 2A). This ensures a complete feedback loop, providing trackability of medication adherence while enhancing coordination among service providers.

All transactions taking place on the PrEPLinker system are designed to be stored as immutable, auditable, and encrypted transactions on a distributed ledger, including being stored on the digital wallet in control of the client (Figure 2B). Local health departments and research institutes could send requests to anonymous clients who hold such information, provide consent and contribute their data for research or fill gaps in existing surveillance records (Figure 2C). With the PrEPLinker platform, if the client decides to comply with the request, they can select all or a subset of the requested data and allow access to that information by the health department or researchers. They also retain the power to withdraw that consent simply by changing their preference in their wallet without having to fill out forms or request action by other organizations.

**Figure 3.**
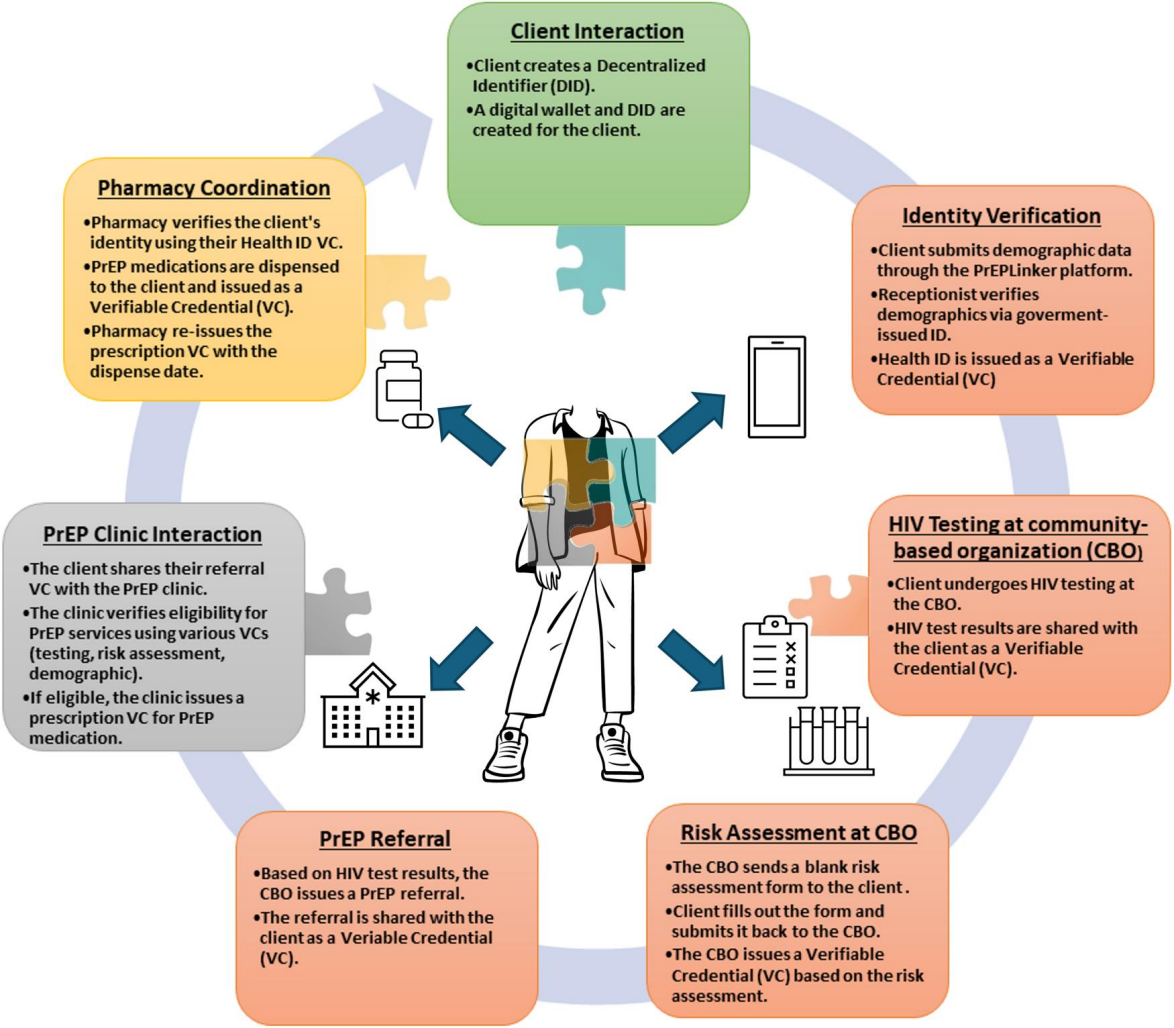
PrEPLinker client workflow. The PrEPLinker client workflow presents a streamlined, client-centered model for PrEP service delivery. In this system, community-based organizations (CBOs), represented by orange boxes, serve as the first point of contact, providing HIV testing and initial risk assessment. Clinical settings, indicated by a grey box, then evaluate clients for PrEP eligibility and issue prescriptions as needed. Finally, a yellow box represents the pharmacies where the medication is dispensed. Here, the client’s digital wallet securely stores personal health information through verifiable credentials (VCs), allowing clients to control their data throughout the care continuum and choosing when and with whom to share information, which enables service providers to coordinate care efficiently while compromising privacy or security.

Figure 3 presents the envisioned PrEPLinker client workflow, illustrating how the system is designed to securely manage and verify client data within a digital wallet system through a client-centered approach to PrEP service delivery.

In this conceptual design, the PrEPLinker workflow envisions starting with the client creating a DID and receiving a digital wallet (green box). The client then visits a CBO (orange box) to submit demographic data for identity verification. A receptionist confirms this information via a government-issued ID, resulting in a Health ID VC. The client then undergoes HIV testing at the CBO, with the test results provided as a VC. Following this, the client completes a risk assessment at the CBO, with results issued as another VC. Based on these assessments, the CBO issues a PrEP referral VC. The client presents this referral at the PrEP clinic (gray box), where eligibility for PrEP services is verified. If eligible, the clinic provides a prescription VC for PrEP medication. Finally, at the pharmacy (yellow box), the client’s identity is verified with the Health ID VC, the medication is dispensed, and the prescription VC is updated with the dispense date.

### PrEPLinker technical architecture

PrEPLinker uses a multitenant architecture hosted on Amazon Web Services (AWS) (Amazon Web Services, Inc., Seattle, WA, United States), supporting both individual and organization wallets. The AWS is a leading cloud service provider and is HIPAA (Health Insurance Portability and Accountability Act) compliant, which means that it can securely store protected health information as required by law. The digital wallets created in this cloud environment and with access only through the personal device of a client or a permissioned organizational representative will interact with the Hyperledger Indy (Hyperledger Foundation, San Francisco, CA, United States) permissioned blockchain framework to store client DIDs and schemas for each credential type.^30^ This means that the middle layer of Hyperledger stores the format and structure of each VC on the shared blockchain platform, but the specific data entry in each record is stored only in the encrypted personal wallet.

**Figure 4.**
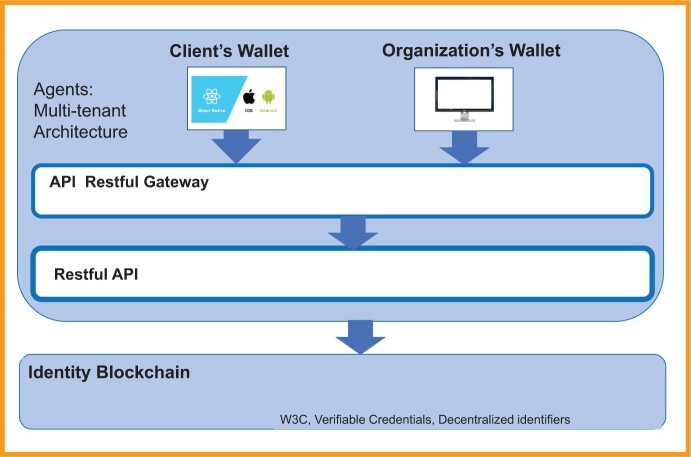
Technical architecture of the PrEPLinker system. The PrEPLinker system architecture uses a multitenant architecture with candidate and organization wallets on the Amazon Web Series (AWS). Hyperledger Aries will be used as a middleware layer (API) to connect the public permissioned Hyperledger Indy layer with the client’s or organization’s digital wallets through a restful API. If this system is required to interact with any other system in addition to the wallets, such as an electronic health record, an additional custom API gateway can be implemented to allow for additional features beyond those of Hyperledger Aries.

Hyperledger Indy was selected over other healthcare-related blockchain platforms because of its focus on client-centric and client-controlled identity and access management, which is essential to our PrEP workflows.^27^^,^^31–33^ The information in the personal wallet is only shared through a secure and trusted connection when the client wants to communicate with an organization. The identity of the client providing this information uses the ledger to establish a secure connection. With a secure and trusted connection, features such as strong privacy guarantees make PrEPLinker a pertinent platform for PrEP services. Hyperledger Aries will be used as a middleware layer (API) to connect the permissioned Hyperledger Indy layer with the client’s or organization’s digital wallets through a restful API. If this system is required to interact with any other system in addition to the wallets, such as an electronic health record, additional custom API Gateways can be implemented to allow for additional features beyond Hyperledger Aries. However, additional interactions were beyond the scope of our current study and will become a future area of development. A simplified version of the architecture with the API-based connection of individual wallets with the permissioned blockchain network is shown in Figure 4.

### Evaluation (preliminary usability)

The descriptive statistics of the 15 participants from the usability evaluation survey are presented in Table 2. The participants were mostly young adults, with 46.7% aged 18-30 years and 40.0% aged 31-45 years. Two-thirds identified as female (66.7%), and the racial and ethnic composition was diverse, with 40.0% identifying as Asian, 33.3% as White, 20.0% as Hispanic or Latino, and 13.3% as African American.

**Table 2.** Participant characteristics for preliminary usability evaluation (*N* = 15).

Characteristics	*N*	%
Age		
18-30 years	7	46.7
31-45 years	6	40.0
46-60 years	2	13.3
Gender identity		
Female	10	66.7
Male	4	26.7
Transgender	1	6.7
Racial/ethnic identity (not mutually exclusive)		
Asian	6	40.0
African American	2	13.3
Hispanic or Latino	3	20.0
White	5	33.3
Other	1	6.7
Prefer not to disclose	1	6.7

The participants demonstrated strong familiarity with health records technology, with all (*N* = 15) regularly using smartphones or computers and 93.3% having visited a healthcare facility in the past year. Additionally, 93.3% had experience using electronic health records, and 26.7% worked in healthcare settings, providing valuable insights.

**Figure 5.**
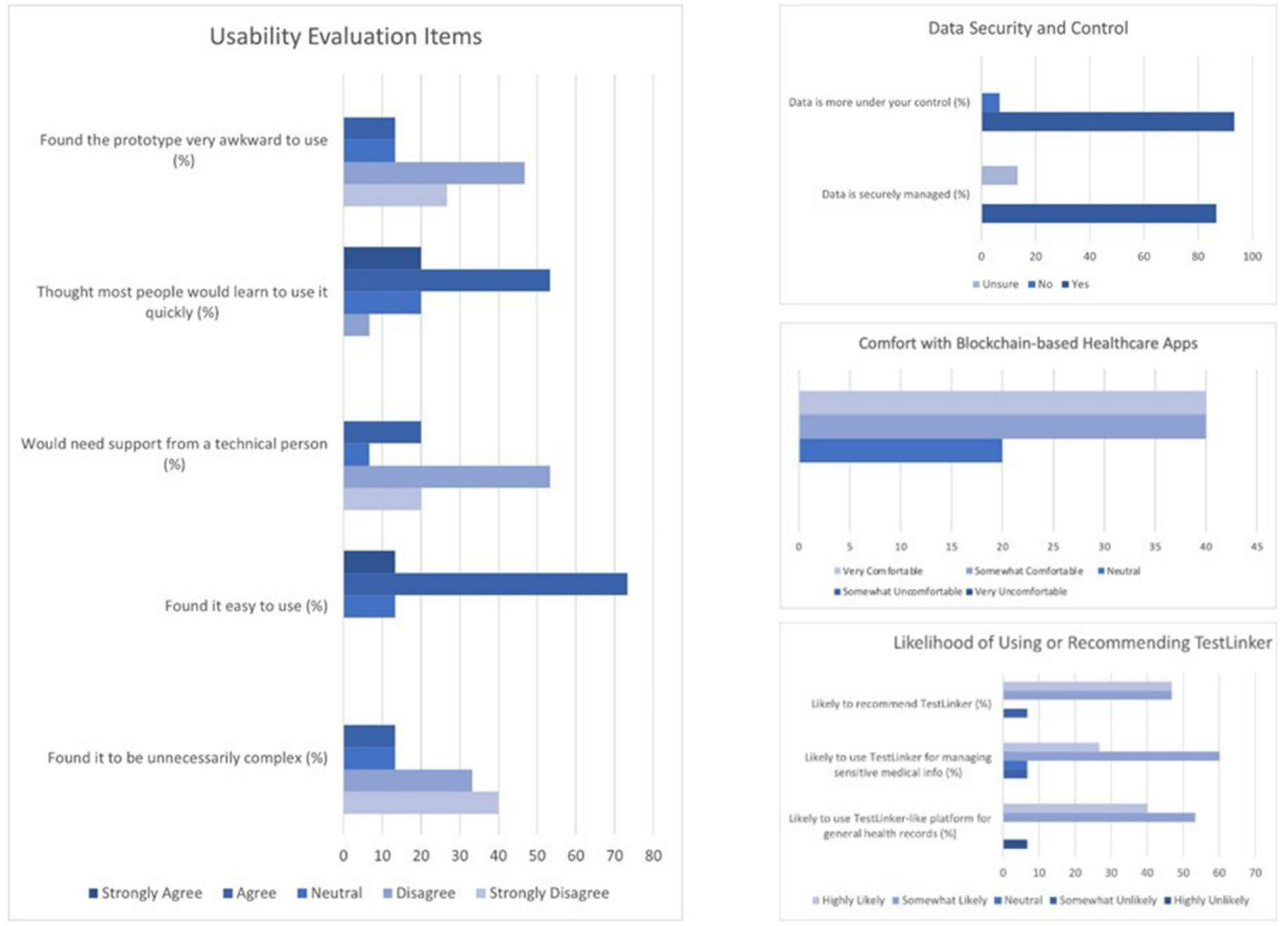
User experience (left) and blockchain impressions of the prototype. The left column presents the results of the usability evaluation for the adjusted PrEPLinker (TestLinker) visual prototype, including ease of use, technical support needs, and overall user-friendliness. The right column presents participants’ impressions of blockchain technology in healthcare, including data security (top), comfort with blockchain applications (right middle), and likelihood of using or recommending the platform (bottom).

Overall, Figure 5 indicates that participants had a positive user experience with the prototype, with most finding it easy to use (86.6%) and not overly complex (73.3%). Similarly, the results indicate that impressions of blockchain technology were favorable, with the majority feeling that their data were securely managed (86.7%) and expressing a willingness to use platforms for both general (93.3%) and sensitive health information (86.7%). More detailed descriptions and results are available in Tables S4 and S5.

## Discussion

The PrEPLinker system is a usable and acceptable technology that addresses several known barriers to PrEP implementation and uptake,^34^ including administrative and program burdens on clients,^35^ mistrust of medical systems and pharmaceutical companies,^36^ and concerns from providers about patient follow-up.^37^ PrEPLinker is designed to aid eligible clients in navigating the complex PrEP care delivery system while ensuring the privacy and security of their personal data through cryptography. It also enables anonymous monitoring of clients who are using PrEP services, strengthening programmatic evaluation.

Leveraging decentralized trust within a P2P network, data sharing across diverse service providers and healthcare entities can help providers monitor individual client retention, and pooled data across organizations may offer new insights to identify gaps in services and overall PrEP trends across a community. Furthermore, PrEPLinker is designed to assist local public health departments and researchers in reaching out to potential PrEP clients; this can be achieved by requesting their consent and information without the necessity of knowing the clients’ identities in advance, thereby protecting client confidentiality. By protecting client privacy and allowing coordinated and auditable workings of PrEP care delivery, these features are particularly beneficial for the most vulnerable populations, those experiencing medical mistrust, and those facing discrimination and stigmatization.

Given the persistent disparities that exist in PrEP uptake, there has been a recent call to monitor metrics of PrEP equity.^38^ PrEPLinker holds potential as a data source to monitor equity and could identify successful organizational pathways to PrEP linkage and retention. The PrEPLinker system is not meant to replace mandatory reporting to health departments for reportable diseases and conditions, but it could supplement public health surveillance records, potentially increasing the completeness and timeliness of reporting. Furthermore, PrEPLinker can operate alongside existing data entry and storage systems at the organization level and is designed to provide the client with a personal record that is complete, secure, and easily shareable if needed for treatment, coordination, reporting, and research.

## Study limitations and directions for blockchain and artificial intelligence integration in HIV prevention

This proof-of-concept study is among the first to design a blockchain-based system for HIV prevention. Although blockchain was introduced in 2008 and operationalized the following year,^39^^,^^40^ its application in US healthcare remains nascent. Blockchain, however, has demonstrated utility in tracking prescriptions, billing, clinical trial data verification, and secure electronic health records.^41^ By contextualizing our findings, we position PrEPLinker as a tool to enhance HIV prevention coordination. Similar systems could improve data liquidity for applications such as managing medical identity and consent and monitoring vaccinations during care transitions.^19^^,^^25^^,^^27^^,^^28^^,^^41–43^ Our preliminary usability testing with a select group of users indicates that PrEPLinker meets the demand for user-friendly blockchain-based healthcare systems, aligning with literature on the importance of accessible design for healthcare professionals and patients.^44^^,^^45^ Studies further suggest that user acceptance of blockchain in healthcare is influenced by factors such as ease of use, perceived security, and transparency.^46^ Within the broader body of research, our findings suggest that PrEPLinker could enhance PrEP care delivery at both the individual and community levels.

Further research is needed to refine PrEPLinker’s technical design and implementation to ensure real-world usability and acceptability. Key priorities include integrating existing electronic health records, embedding real-time social determinants of health data into providers’ workflows,^47^ and using health information exchanges as an added source of community-wide data. Blockchain technology in healthcare faces challenges, such as building P2P shared infrastructure and gaining community-wide adoption and trust, especially in a highly regulated environment. The gap between blockchain development and implementation often limits its impact to the proof-of-concept stage, emphasizing the need for robust testing in real-world settings.

Future research on PrEPLinker should evaluate its feasibility, acceptability, effectiveness, and cost efficiency to support its further development and potential scale-up. The suggested studies include focus groups^45^ to identify adoption barriers, collaborative evaluations to assess provider involvement,^48^^,^^49^ effectiveness-implementation hybrid designs^50^ to compare PrEPLinker to standard care, and a cost-effectiveness analysis^51^ to guide evidence-based improvements.

Integrating PrEPLinker with advanced machine learning methodologies such as domain adaptation and explainable artificial intelligence (AI)^52^ enables PrEPLinker to adapt to various social, cultural, and operational contexts across other cities and jurisdictions. Explainable AI can also generate interpretable outputs, providing policymakers with actionable insights into community needs and resource allocations coupled with ensuring trust and transparency through immutable records. Graph neural networks have shown promise in improving HIV risk prediction through multilayered social network data,^53^ identifying high-risk individuals and patterns to inform personalized care and targeted intervention.^54^ This approach enables PrEPLinker to securely allocate resources and tailor care on the basis of each client’s social context and risk factors through its decentralized identity (DID) and VCs. By detecting patterns in social networks, PrEPLinker can more effectively direct resources and personalize HIV prevention interventions, supporting optimized PrEPLinker care delivery in alignment with EHE goals.

Blockchain can also improve the system by verifying the integrity of AI-generated tools, ensuring that the data are trusted and verifiable. Current blockchain systems use permissioned governance, where trusted entities set rules for verifying, validating, and accessing information.^55^ This decentralized model reduces the reliance on a small number of major organizations, which can be particularly beneficial during national emergencies such as COVID-19 (coronavirus disease 2019), by promoting a balanced distribution of resources and information among community collaborators.^22^

PrEPLinker shows potential promise for benefiting clients, providers, and public health officials by delivering actionable insights that support practitioners and inform policymakers. For evaluation purposes, it aids in tracking patterns of successful (or unsuccessful) linkage and retention, enables providers to demonstrate client linkage success to funders, and supports the monitoring of equitable PrEP access. Through an opt-in model, clients can receive appointment reminders, access other available services, and help fill gaps in public health surveillance. PrEPLinker also strengthens continuity of care, allowing providers to track referrals and monitor ongoing treatment for other conditions (eg, STDs, vaccination). For clients, PrEPLinker reduces administrative burdens and provides consent management and data control over their personal health records. Our vision for HIV prevention is embodied in the design of PrEPLinker, a system envisioned to make PrEP care easier for clients, more efficient for providers, and more effective across the PrEP care continuum.

## Conclusion

In conclusion, our proposed blockchain-based PrEPLinker design has the potential to streamline workflows and facilitate the secure sharing of data throughout the local PrEP care continuum. We hope that this client-centric and community-engaged innovative platform design can advance PrEP care delivery within the emerging Web3 ecosystem. This leads to more equitable healthcare access, increasing HIV testing, and supporting PrEP uptake and adherence, especially for the most vulnerable populations and underserved communities in the effort toward the elimination of HIV.

## Supplementary Material

ooae140_Supplementary_Data
