# Supplementary material for: Designing a blockchain technology platform for enhancing the pre-exposure prophylaxis care continuum
Source: JAMIA Open. 2024 Dec 19;7(4):ooae140. doi: 10.1093/jamiaopen/ooae140 (PMC11658693; doi:10.1093/jamiaopen/ooae140)
Supplement: ooae140_Supplementary_Data [file ooae140_supplementary_data.docx]

# SUPPLEMENTAL MATERIALS

## INTERVIEW GUIDE FOR COMMUNITY COLLABORATORS

Table S1 presents the interview guides used for 12 key informant interviews with local PrEP collaborators to assess their current workflows and barriers experienced in delivering or receiving PrEP services.

**Table S1**: Interview guide questions for Houston-area PrEP collaborators by organizational type

| **Stakeholder** | **Interview Questions** |
| --- | --- |
| **CBO (Community-Based Organization)** | 1. How do patients get referred to (or find out about) your organization?  2. How does your staff determine eligibility for the PrEP program?  3. What are your current workflows and referral processes for PrEP patients?  4. What patient data do you collect from patients entering the PrEP program?  5. How does your staff refer patients to the PrEP program?  6. Do you need to track the completion of patient referrals?  7. How does your organization track or “close the loop” on patients throughout the PrEP program?  8. How do you handle the sensitivity and privacy related to patients’ HIV status?  9. What data problems and workflow issues do you encounter?  10. What data do you contribute or report to the Houston Health Department? |
| **PrEP Provider** | 1. How do patients get referred to (or find out about) your organization?  2. How does your staff determine a patient’s eligibility for the PrEP program?  3. What are your current workflows for PrEP patients?  4. After a patient is referred to you for PrEP, what steps does the patient experience?  5. What patient data do you collect or need from patients entering the PrEP program?  6. How does your organization track or “close the loop” on patients throughout the PrEP program?  7. Do you need to track referrals and prescription fulfillment?  8. Have you encountered issues with patients picking up prescriptions? How do you know if prescriptions are filled or not?  9. How do you handle the sensitivity and privacy related to HIV+ patients?  10. What data problems and workflow issues do you encounter?  11. What data do you contribute or report to the Houston Health Department? |
| **Pharmacy** | 1. How do patients come to your pharmacy?  2. What are your current workflows for PrEP patients?  3. What steps does the patient experience?  4. What patient data do you collect or need from patients picking up PrEP prescriptions?  5. How does your organization track or “close the loop” on patients throughout the PrEP program?  6. Have you encountered issues with patients picking up prescriptions or monitoring compliance?  7. Do you track compliance and send information to providers?  8. What happens to unpicked medications?  9. How do you handle the sensitivity and privacy related to HIV+ patients?  10. What data problems and workflow issues do you encounter?  11. What data do you contribute to the Houston Health Department? |

## DATA AND METHODS AT EVALUATION PHASE

The PrEPLinker system was designed to address barriers to accessing pre-exposure prophylaxis (PrEP) for individuals who have tested negative for HIV. Between Fall 2023 and Spring 2024, we recruited 15 students and employees at the [Institution name blinded for review] through email listservs and personal contacts via research team members based at that institution.

Data collected during this period were part of Phase II of the TestLinker project, titled “*Blockchain-Based HIV Testing Management System*” (HSC-SPH-22-0567; IRB amendment approved on 01/03/2024) funded through an Ending the HIV Epidemic (EHE) pilot supplement (P30AI161943).

PrEPLinker was designed based on the MediLinker user interface (UI). Building on the PrEPLinker’s design, TestLinker extended the scope of the blockchain-enabled platform by adopting an HIV status-neutral approach, encompassing workflows for both individuals living with and without HIV. The TestLinker project uses a mix-method approach and helps individuals manage and share their HIV test results and health data with providers across healthcare settings, regardless of their HIV status. We pilot-tested the TestLinker UI and its prototypes using Figma prototyping to assess feasibility, user satisfaction, and acceptability. Table S2 presents features of the three digital wallet systems.

**Table S2:** Digital wallet systems comparison (proof-of-concept stage)

| Feature | MediLinker | PrEPLinker | TestLinker |
| --- | --- | --- | --- |
| Development timeline | Initial system implemented (2019-2023) | Built on MediLinker (2021-2024) | Extended from PrEPLinker (2022-2024) |
| Primary aim | Patient-centric healthcare data management for transitions of care | PrEP service coordination and data management | HIV testing data management with status-neutral approach |
| Targeted users | Healthcare providers and patients | PrEP services, pharmacies, labs, social services | Black and Latinx sexual minority men, healthcare providers |
| Location | Austin, TX | Houston area, TX | Houston area, TX |
| Evaluation methods | Domain expert assessment of clinical, organizational, regulatory, ethical, and social issues  Usability testing with Pilot testing 69 participants | Community collaborator interviews  Usability testing with 15 participants (shared with TestLinker) | Focus groups with providers (CFIR framework)  Focus groups with intended population  Usability testing with 15 participants |
| Key findings | Blockchain feasible for patient-centric approach  Faced institutional buy-in challenges | Complex referral process identified  Need for community learning collaborative  Collaborator buy-in needed | Completed assessment of blockchain acceptability, manuscript in progress |
| Project status | Proof-of-concept, design validated through evaluation | Proof-of-concept, design validated through TestLinker evaluation | Proof-of-concept, design validated through evaluation |
| Contribution | Established baseline blockchain framework for identity and healthcare data management | Developed conceptual workflows for PrEP service coordination | Proposed HIV status-neutral approach in system design |

## Procedures

Participants were selected to represent a general health-seeking population, with no eligibility criteria other than age (needed to be at least 18 years old). Using purposive sampling, we aimed to include a diverse range of ages and racial/ethnic identities. No identifying information was captured in the workflow for the study, and the study was classified as non-human subjects research by the [University] Institutional Review Board, so written informed consent form was not required.

Prior to interacting with participants, our team developed a visual representation of upstream PrEP clinical workflows based on the designs and insights from key informant interviews, using Figma (Figma Inc), an interface design tool. This study involved simulated test scenarios, including: 1) setting up digital IDs, 2) receiving HIV test results, 3) filling in a risk assessment, and 4) receiving a PrEP referral. During a 60-minute virtual session conducted by author DTH over Zoom, participants interacted with Figma designs, performing the four tasks listed above as an avatar named “John Doe” and using synthetic PrEP data.

Participants were provided with a description of the study and data usage, and they were free to decline participation without explanations or penalty. A letter of information was given to each participant, and downloading and setting up the application was considered as consent to participate in the study. Sessions were recorded by a researcher, and following the interaction with the TestLinker application, participants completed an online usability survey via REDCap. Each participant received a $20 gift card as an incentive. Survey data were analyzed descriptively.

## Survey Questions

Our evaluation survey was composed of the following four sections.

**Table S3:** Survey questions on demographics, usability, and blockchain technology

| **Category** | **Question** | **Response Options** |
| --- | --- | --- |
| **Participant Demographic** | | |
|  | *1. Please select the age group you fall under* | Under 18  18-30  31-45  46-60  Over 60 |
|  | *2. What gender do you identify with?* | Female  Male  Transgender  Non-binary  Choose not to disclose |
|  | *3. Please select your ethnicity.* | American Indian or Alaska Native,  Asian,  African American,  Hispanic or Latino,  Native Hawaiian or Other Pacific Islander,  White,  Other,  Choose not to disclose |
|  | *4. Do you own and regularly use a smartphone or personal computer?* | Yes  No |
|  | *5. Are you working in a healthcare facility as healthcare personnel?* | Yes  No |
|  | *6. Have you visited a clinic or health facility as a patient in the last year?* | Yes  No |
|  | *7. Have you previously used an electronic health record system?* | Yes  No |
| **Prototype Usability Questions** | | |
|  | *1. Did you find TestLinker to be unnecessarily complex?* | Strongly Disagree  Disagree  Neutral  Agree  Strongly Agree |
|  | *2. Did you find TestLinker was easy to use?* | Strongly Disagree  Disagree  Neutral  Agree  Strongly Agree |
|  | *3. Did you think you would need the support of a technical person to use TestLinker?* | Strongly Disagree  Disagree  Neutral  Agree  Strongly Agree |
|  | *4. Did you think that most people would learn to use TestLinker very quickly?* | Strongly Disagree  Disagree  Neutral  Agree  Strongly Agree |
|  | *5. Did you find the TestLinker prototype very awkward to use?* | Strongly Disagree  Disagree  Neutral  Agree  Strongly Agree |
| **Impressions of Blockchain Usage in Healthcare** | | |
|  | *1. Do you feel your data is securely managed with TestLinker’s patient-centric model?* | Yes  No  Unsure |
|  | *2. Do you feel your data is more under your control with TestLinker’s patient-centric model?* | Yes  No  Unsure |
|  | *3. How comfortable are you using blockchain-based applications for healthcare management?* | Very uncomfortable  Somewhat uncomfortable  Neutral  Somewhat comfortable  Very comfortable |
|  | *4. How likely are you to use a TestLinker-like platform for your general health records?* | Highly unlikely  Somewhat unlikely  Neutral  Somewhat likely  Highly likely |
|  | *5. How likely are you to use TestLinker again for managing highly sensitive medical information like HIV status?* | Highly unlikely  Somewhat unlikely  Neutral  Somewhat likely  Highly likely |
|  | *6. How likely are you to recommend TestLinker to someone with a highly sensitive health condition?* | Highly unlikely  Somewhat unlikely  Neutral  Somewhat likely  Highly likely |

Reference: U.S. General Services Administration. (Accessed on October 29, 2024). Usability. Digital.gov. <https://digital.gov/topics/usability/#:~:text=Usability%20Evaluation%20focuses%20on%20how,related%20to%20a%20new%20site>.

## Evaluation Survey Results

**Table S4** presents the results of user experience with the TestLinker visual prototype, showing that participants generally found the system easy to use and not overly complex.

**Table S4:** Results of user experience with visual prototype (TestLinker)

| *Did you find TestLinker to be unnecessarily complex?* | *N* | *%* |
| --- | --- | --- |
| Strongly disagree | 6 | 40.0% |
| Disagree | 5 | 33.3% |
| Neutral | 2 | 13.3% |
| Agree | 2 | 13.3% |
| Strongly agree | 0 | 0.0% |
| *Did you find TestLinker was easy to use?* |  |  |
| Strongly disagree | 0 | 0.0% |
| Disagree | 0 | 0.0% |
| Neutral | 2 | 13.3% |
| Agree | 11 | 73.3% |
| Strongly agree | 2 | 13.3% |
| *Did you think you would need the support of a technical person to use TestLinker?* | |  |
| Strongly disagree | 3 | 20.0% |
| Disagree | 8 | 53.3% |
| Neutral | 1 | 6.7% |
| Agree | 3 | 20.0% |
| Strongly agree | 0 | 0.0% |
| *Did you think that most people would learn to use TestLinker very quickly?* | |  |
| Strongly disagree | 0 | 0.0% |
| Disagree | 1 | 6.7% |
| Neutral | 3 | 20.0% |
| Agree | 8 | 53.3% |
| Strongly agree | 3 | 20.0% |
| *Did you find the TestLinker prototype very awkward to use?* |  |  |
| Strongly disagree | 4 | 26.7% |
| Disagree | 7 | 46.7% |
| Neutral | 2 | 13.3% |
| Agree | 2 | 13.3% |
| Strongly agree | 0 | 0.0% |

Most participants (73.3%, N=15) disagreed that the prototype was unnecessarily complex, while 86.6% found it easy to use. 73.3% indicated they did not need technical support, and 73.3% believed most users would quickly learn to navigate the prototype. The majority (73.4%) disagreed that it was awkward to use.

**Table S5:** Results of impressions of blockchain in healthcare

| *Do you feel your identifying and medical data is securely managed with its patient-centric model?* | *N* | *%* |
| --- | --- | --- |
| Yes | 13 | 86.7% |
| No | 0 | 0.0% |
| Unsure | 2 | 13.3% |
| *Do you feel your identifying and medical data is more under your control with its patient-centric model?* |  |  |
| Yes | 14 | 93.3% |
| No | 1 | 6.7% |
| Unsure | 0 | 0.0% |
| *How do you feel using blockchain-based applications for healthcare management* |  |  |
| Very uncomfortable | 0 | 0.0% |
| Somewhat uncomfortable | 0 | 0.0% |
| Neutral | 3 | 20.0% |
| Somewhat comfortable | 6 | 40.0% |
| Very comfortable | 6 | 40.0% |
| *How likely would you use a TestLinker-like platform for your general health records?* |  |  |
| Highly unlikely | 1 | 6.7% |
| Somewhat unlikely | 0 | 0.0% |
| Neutral | 0 | 0.0% |
| Somewhat likely | 8 | 53.3% |
| Highly likely | 6 | 40.0% |
| *How likely would you use TestLinker again for managing your highly sensitive medical information such as HIV status?* |  |  |
| Highly unlikely | 0 | 0.0% |
| Somewhat unlikely | 1 | 6.7% |
| Neutral | 1 | 6.7% |
| Somewhat likely | 9 | 60.0% |
| Highly likely | 4 | 26.7% |
| *How likely would you recommend TestLinker to someone you know with a highly sensitive health condition?* |  |  |
| Highly unlikely | 0 | 0.0% |
| Somewhat unlikely | 1 | 6.7% |
| Neutral | 0 | 0.0% |
| Somewhat likely | 7 | 46.7% |
| Highly likely | 7 | 46.7% |

86.7% felt their data was securely managed, and 93.3% believed the client-centric model gave them more control over their health information. 80% were comfortable using blockchain-based applications. A majority (93.3%) were likely to use the platform for general health records, with 86.7% willing to use it for managing sensitive medical information, such as HIV status. 93.4% were likely to recommend the system to others with sensitive health conditions.
